# Evaluation of the Efficacy of Intravenous Push and Intravenous Piggyback Ceftriaxone in Critically Ill Patients

**DOI:** 10.3390/antibiotics13100921

**Published:** 2024-09-26

**Authors:** Elly R. Sherman, Nha Hue Ta, Trisha N. Branan, Natt Patimavirujh, Luren Ashton Dickinson, Christopher M. Bland, Susan E. Smith

**Affiliations:** 1 Department of Clinical and Administrative Pharmacy, University of Georgia College of Pharmacy, Athens, GA 30602, USA; elly.sherman@vumc.org (E.R.S.); nha.ta@uga.edu (N.H.T.); tbranan@uga.edu (T.N.B.); ladickinson@uga.edu (L.A.D.); 2 Department of Clinical and Administrative Pharmacy, University of Georgia College of Pharmacy, Savannah, GA 31405, USA; npatimavirujh@tgh.org (N.P.); cmbland@uga.edu (C.M.B.)

**Keywords:** ceftriaxone, critical illness, critical care, administration, intravenous, pharmacokinetics, antibiotics, beta-lactam

## Abstract

**Background/Objective**: Intravenous fluid shortages have led to fluid-sparing measures such as intravenous push (IVP) administration of antibiotics. This study aimed to compare the safety and efficacy of IVP and intravenous piggyback (IVPB) ceftriaxone in critically ill patients. **Results**: Demographics were similar in IVP (*n* = 201) and IVPB (*n* = 200) groups. Sequential Organ Failure Assessment (SOFA) score was higher, and sepsis and septic shock were more common in the IVP group. Treatment failure occurred in 37.8% of IVP and 19.5% of IVPB groups (*p* < 0.001). Hospital mortality was more common with IVP (21.4% vs. 9.5%, *p* < 0.001). Hospital LOS was longer with IVP while intensive care unit (ICU) LOS was similar between the groups. IVP ceftriaxone (OR 2.33, 95% CI 1.43–3.79) and the SOFA score (OR 1.18, 95% CI 1.1–1.27) were associated with treatment failure. **Methods**: A single-center, retrospective cohort study included adults admitted to an ICU from 2016 to 2021 who received empiric ceftriaxone for ≥72 h. The primary outcome was treatment failure, defined as a composite of inpatient mortality or escalation of antibiotics. Secondary outcomes included length of stay (LOS) and mortality. Chi-squared and independent-sample *t*-tests were used. Treatment failure was evaluated using multivariate logistic regression. **Conclusions**: Compared to IVPB, IVP ceftriaxone was associated with higher treatment failure in critically ill patients. Both safety and efficacy should be considered before implementing novel antibiotic administration strategies in practice based primarily on convenience.

## 1. Introduction

Due to its once-daily dosing schedule, spectrum of activity, and lack of renal or hepatic dose adjustment for the majority of patients, ceftriaxone is commonly used in patients admitted to the critical care setting for community-acquired infections [1]. By inhibiting the cell wall synthesis of susceptible pathogens, ceftriaxone functions as a bactericidal agent with activity against many common Gram-positive and Gram-negative pathogens, such as *Streptococcus pneumoniae* and *Escherichia coli* [2]. Intravenous piggyback (IVPB) has been the mainstay route of administration for ceftriaxone in the hospital setting; however, due to IV fluid shortages brought on by Hurricane Maria, which were further exacerbated by the COVID-19 pandemic, IV push (IVP) administration has been implemented into routine practice by many institutions [3,4]. Concern for IVP beta-lactams may be the result of their time-dependent activity and concentrations dropping below minimum inhibitory concentrations earlier in the dosing interval, leading to clinical failure, especially in a critically ill population with significant pharmacokinetic/pharmacodynamic barriers to appropriate free drug concentrations. Ceftriaxone has a longer half-life of 5.8 to 8.7 h compared to other beta-lactams, potentially allowing for adequate exposure time to provide microbiologic and clinical cure even with IVP administration [2]. The availability of IVP ceftriaxone as a safe and effective treatment option may provide beneficial clinical and economic outcomes and avoid compatibility issues that are common within a critically ill patient on numerous IV medications.

When preparing ceftriaxone for IV infusion, each vial must be reconstituted with normal saline, dextrose 5% in water, or sterile water for injection, with further dilution in a larger fluid bag required for IVPB administration [5]. Therefore, administration via IVP supports fluid stewardship efforts by requiring only the initial fluid used for reconstitution. Notably, antibacterial agents often account for the largest fluid administration volumes and frequencies in ICU patients [6]. IVP administration of any medication can be associated with a potential for adverse events such as vascular trauma, infiltration, and phlebitis due to the greater concentration of drugs administered over a shorter period of time. Specifically, rapid administration of ceftriaxone via the IVP route has resulted in case reports of palpitations and tachycardia, diaphoresis, shivering, and restlessness [5]. Primary research evaluating this administration route within hospitalized patients suggests no complications; however, studies are limited and span many years [7,8,9]. This preliminary safety evidence supports the use of IVP ceftriaxone as a viable treatment option to be evaluated within the critical care setting.

Previous studies have examined safety parameters but have not evaluated the impact of ceftriaxone administration strategy on patient outcomes in the critical care setting. The primary purpose of this study was to evaluate the impact of IVPB and IVP administration of ceftriaxone on the clinical outcomes of critically ill patients. It was hypothesized that IVPB and IVP administration of ceftriaxone would have similar efficacy in critically ill patients.

## 2. Results

In total, 401 adult patients were included in the empiric treatment portion of the study, comprising 201 patients in the IVP group and 200 patients in the IVPB group. Demographics of this primary analysis were similar between the IVP and IVPB groups and included predominantly white race (IVP 70% vs. IVPB 74%), majority male patients (58% vs. 54%), and an average age of 61 years for both groups (Table 1). The mean Sequential Organ Failure Assessment (SOFA) score at ceftriaxone initiation was significantly higher in the IVP group (6.4 vs. 5.4, *p* = 0.002). Sepsis and septic shock at the time of ceftriaxone initiation were also more common in the IVP group (sepsis: 56% vs. 31%, *p* < 0.001; septic shock: 29% vs. 11%, *p* < 0.001).

The most common source of infection in both groups was respiratory (53% vs. 40%, *p* = 0.023). Urinary tract infections, severe infections, and other/unknown sources were more common in the IVPB group, while intra-abdominal infections were more prevalent in the IVP group. Notably, 110 patients (55%) in the IVP group and 130 (65%) in the IVPB group had a positive culture result (*p* = 0.041). The pathogens isolated from cultures included *Escherichia coli* (29% of positive cultures), *Staphylococcus aureus* (19%), *Klebsiella pneumoniae* (15%), *Streptococcus pneumoniae* (10%), *Haemophilus influenzae* (9%), and other pathogens occurring at a frequency of less than four counts each (16%).

Interventions, including duration of ceftriaxone, average daily dose, and the total duration of antibiotics, were similar between the two groups (Table 1).

### 2.1. Outcomes

In this empiric therapy cohort, the primary composite outcome of treatment failure (inpatient mortality or escalation of antibiotic therapy due to worsening clinical status) occurred in 38% of the IVP group and 20% of the IVPB group (*p* < 0.001) (Table 2). All-cause hospital and ICU mortality were more common in the IVP group (hospital: 21% vs. 10%, *p* < 0.001; ICU: 17% vs. 9%, *p* = 0.013), as was the rate of antibiotic escalation (25% vs. 12%, *p* < 0.001) (Table 2). Hospital length of stay (LOS) was longer in the IVP group, while ICU LOS was similar between the groups. After controlling for potentially confounding variables, IVP ceftriaxone (odds ratio [OR] 2.33, 95% confidence interval [CI] 1.43–3.79) and the SOFA score at ceftriaxone initiation (OR 1.18, 95% CI 1.1–1.27) were independent predictors of treatment failure, while longer duration of ceftriaxone therapy (OR 0.86, 95% CI 0.78–0.96) was associated with decreased odds of treatment failure (Table 3).

### 2.2. Sensitivity Analyses

In the first sensitivity analysis excluding all patients with a potentially ceftriaxone-intermediate or ceftriaxone-resistant pathogen (IVP: *n* = 176, IVPB: *n* = 178), baseline characteristics were similar to the overall cohort. There were no statistically significant differences in demographic data or baseline characteristics (Table 4). Similar to the larger cohort, the mean SOFA score at the initiation of therapy was higher in the IVP group (6.4 vs. 5.3, *p* < 0.001), as were rates of sepsis and septic shock (sepsis: 56% vs. 29%, *p* < 0.001; septic shock: 30% vs. 10%, *p* < 0.001). Intervention data were similar between the groups (Table 4). The second sensitivity analysis retaining patients with *Citrobacter* or *Enterobacter* species infections included 184 patients in the IVP group and 183 patients in the IVPB group and followed the same trends (Table 4).

In both sensitivity analyses, outcomes were similar to the primary analysis with treatment failure occurring at a higher rate in the IVP group (*p* < 0.001 for both). The individual components of the primary outcome, escalation of antibiotic therapy and all-cause inpatient mortality, also occurred at a higher rate in the IVP group in both analyses (Table 5). In multivariate binary logistic regression models controlling for potentially confounding variables, IVP administration of ceftriaxone and the SOFA score were retained as independent predictors of treatment failure (Table 6).

## 3. Discussion

In this retrospective, observational cohort study of critically ill patients, IVP administration of ceftriaxone was associated with a higher rate of treatment failure compared to IVPB administration in both the empiric treatment analysis and sensitivity analyses. Additionally, the escalation of antibiotic therapy and mortality were both more frequent in the IVP groups. These findings could potentially be explained by the lower infusion time in IVP administration. Since the efficacy of beta-lactam antibiotics is directly related to the amount of time during which concentrations are above the minimal inhibitory concentration, ceftriaxone’s optimal pharmacodynamics might be limited by the shortened infusion time when given via IVP [10].

Previous studies related to IVP ceftriaxone administration have largely been in the setting of emergency departments. Multiple studies have shown that IVP ceftriaxone is safe and effective when administered as a first dose in the emergency department as well as for outpatient parenteral antimicrobial therapy [7,10,11]. However, there are limited data on IVP use in critically ill patients. Monte Carlo simulations of 30 min and 5 min infusions of meropenem, cefepime, and aztreonam based on data from healthy volunteers and those with active infections found minimal to no effect on pharmacodynamic exposures when administering these beta-lactams by IVP over 5 min [12]. In a clinical, real-world setting, however, IVP administration of cefepime was observed to be associated with increased treatment failure in a critically ill population [13]. This finding was not replicated in non-critically ill patients [14]. The elimination half-life of cefepime is approximately 2 h, significantly shorter than ceftriaxone; yet, worse outcomes were found with IVP administration in both studies [14,15]. Furthermore, the recent ACORN trial that randomized critically ill patients to piperacillin/tazobactam and cefepime found a decreased number of days alive and free from delirium/coma in the cefepime group. However, the study is criticized for its use of IVP cefepime versus extended infusion piperacillin/tazobactam, bringing increased attention to this question of beta-lactam administration strategy [16].

Ceftriaxone is significantly protein-bound, which leads to its once-daily dosing for most clinical indications [2]. Critically ill patients with hypoalbuminemia may be more affected by pharmacokinetic/pharmacodynamic dosing, leading to suboptimal free drug concentrations of ceftriaxone for antibacterial activity. Ertapenem, another commonly used long-half-life beta-lactam, was found in an observational study to have a five-fold increased risk of mortality when treating Enterobacterales infections compared to other carbapenems in patients who had hypoalbuminemia (<2.5 g/dL) [17]. A recent Infectious Diseases Society of America panel recommended using meropenem or imipenem/cilastatin rather than ertapenem for patients with extended-spectrum beta-lactamase *E. coli* infections in hypoalbuminemic patients [18].

While the findings of this study support the preferential use of IVPB ceftriaxone, there are additional factors to be considered when selecting a route of administration. IVP administration of medications may be preferred in certain patient populations where fluid overload is a concern, such as congestive heart failure. Notably, dual-chamber IV bags of ceftriaxone for IVPB administration are available in both 1 g and 2 g formulations, each with a total volume of 50 milliliters [19]. While not FDA-approved, IVP administration has been shown to be safe and effective when ceftriaxone is reconstituted with 10 milliliters of sterile water for injection for both 1 g and 2 g doses [5]. Over a 7-day treatment course for bacterial meningitis, for example, IVP dosing could reduce fluid administration by 560 milliliters when dosed at 2 g every 12 h. Minimizing IV fluid administration may be a compelling reason to consider IVP over IVPB in select patients.

In addition to being fluid-sparing, IVP administration also decreases the burden on pharmacy and nursing staff when dispensing and administering antibiotics. Nurses can administer medications separately, without having to contend with IV compatibilities and stagger infusion times. Cost analyses of similar antibiotics, including cefazolin, have shown significant cost savings with IVP administration [20]. However, these advantages do not negate the potential increased risk of clinical failure and mortality, which outweigh any potential operational benefit or fluid offset.

Ceftriaxone has been shown to exhibit higher volumes of distribution and increased drug clearance in critically ill patients with normal renal function [21]. More specifically, augmented renal clearance (ARC), which is defined by a creatinine clearance of 150 mL/min or greater, is associated with an increased risk of empiric ceftriaxone under-dosing due to subtherapeutic plasma concentrations [21]. Higher blood concentrations and therapeutic drug monitoring may be needed to prevent therapeutic failure in critically ill patients with ARC receiving ceftriaxone.

Further, a recent retrospective study comparing ceftriaxone dosing in ICU patients without central nervous system infection showed that patients treated with ceftriaxone 2 g per day were significantly less likely to experience treatment failure than those given 1 g per day [22]. In the current study, the average daily dose was 1.33 g in the IVP group and 1.40 g in the IVPB group (similar to the sensitivity analyses). It is possible that these lower average daily doses contributed to higher treatment failure rates in both groups.

The definition of treatment failure used in this study (inpatient mortality and/or escalation of antibiotic therapy due to worsening clinical status) has been used in similar studies [13,22]. This definition is designed to capture instances where ceftriaxone treatment was unsuccessful. However, it does not account for the exact reason for the escalation of antibiotic therapy. For example, a patient may have acquired a nosocomial infection during their stay that requires an antimicrobial with broadened coverage. This would not necessarily be a case of “treatment failure” because the original infection may have been successfully treated with ceftriaxone. Additionally, inpatient mortality did not always occur as a direct result of the patient’s infection. A patient with severe trauma started on ceftriaxone for a potential wound infection may expire as a result of blood loss and hemodynamic instability, rather than infection. Therefore, the true rate of ceftriaxone treatment failure may be greater or smaller than what is reported.

A sensitivity analysis was performed to exclude patients with potentially non-susceptible pathogens, as the inclusion of these patients could have confounded the results. A second sensitivity analysis retained patients whose cultures grew *Citrobacter* or *Enterobacter* species, as there is a possibility of inducible ceftriaxone resistance by these pathogens [23]. A recent panel recommends avoiding ceftriaxone within invasive infections caused by organisms at moderate risk of significant AmpC production, such as *Citrobacter freundii*, *Klebsiella aerogenes* (formerly *Enterobacter aerogenes*), or *Enterobacter cloacae*, even if an isolate initially tests susceptible to them, due to this concern, but some studies have demonstrated similar outcomes to non-third-generation cephalosporins [18,19,24,25]. The rates of potential pathogens not susceptible to ceftriaxone were similar between the IVP and IVPB groups, as were all outcomes.

Limitations include the retrospective, single-center design, lack of demographic diversity, and higher incidence of sepsis and septic shock in the IVP group. Additionally, some potentially confounding variables such as comorbidities, concomitant medications, and viral coinfections were not collected. With less than 2% of patients being of Hispanic origin and less than 1% of Asian origin, it may be difficult to extrapolate the findings of this study to more diverse patient populations. Moreover, patients receiving IVP ceftriaxone had a higher incidence of sepsis and septic shock, as well as a higher SOFA score. This difference in the baseline severity of illness is a limitation of the study; however, the relationship between treatment failure and IVP administration of ceftriaxone was maintained in binary logistic regression controlling for the difference in SOFA scores between the two groups. Since the SOFA score is directly related to sepsis/shock and is more comprehensive, sepsis and septic shock were not included in the regression to prevent collinearity. However, it may not be possible to fully separate the primary outcome from the severity of illness of this group. A possible reason for the difference in the severity of illness between the groups could be the greater emphasis placed on antimicrobial stewardship in recent years, coinciding with the time period in which IVP ceftriaxone was used, as well as higher patient acuity in more recent years. Providers may have become more comfortable using a comparatively narrower-spectrum antibiotic for patients with sepsis or septic shock without risk factors for *Pseudomonas aeruginosa*, leading to more use of ceftriaxone with higher severity of illness in the more recent years of the study. Despite these limitations, findings suggest a possible benefit with IVPB administration of ceftriaxone in ICU patients.

Opportunities for future research include comparisons of IVP and IVPB ceftriaxone for specific disease states such as meningitis or pneumonia to gain a better understanding of the effects of IVP administration at the specific doses for these indications. Research in different populations including non-ICU and pediatric patients is also warranted. Finally, confirmation through a prospective, randomized study design would help corroborate the findings of this study.

## 4. Materials and Methods

A single-center, retrospective, observational cohort study was conducted at a 427-bed community teaching hospital. The study was approved by the Piedmont Healthcare Institutional Review Board (Atlanta, GA, USA) as exempt research before data collection began. The STROBE Cohort reporting guidelines were followed. Adult patients admitted to the ICU for at least 72 h between March 2016 and January 2021 who received empiric ceftriaxone for at least 72 h were included, until a convenience sample of 200 patients in each group was achieved. Doses of ceftriaxone administered in the emergency department were included in the minimum duration of 72 h, provided the patient was admitted directly to the ICU. The hospital system transitioned from IVPB ceftriaxone administration to IVP in May 2018 as part of a system-wide initiative to conserve small-volume parenteral products. Therefore, patients admitted between March 2016 and April 2018 received IVPB administration, and those admitted between May 2018 and January 2021 received IVP administration. Patients were excluded from the study on the basis of receipt of both IVP and IVPB ceftriaxone as well as pregnancy. The primary analysis evaluated ceftriaxone as empiric therapy, regardless of the pathogen(s) isolated. Two sensitivity analyses were conducted to exclude patients with infections known to be caused by potentially non-susceptible pathogens. The first sensitivity analysis excluded patients with a culture positive for *Enterococcus*, *Enterobacter*, and *Citrobacter* species, as well as methicillin-resistant *Staphylococcus aureus*, *Pseudomonas aeruginosa*, or *Stenotrophomonas maltophilia*. Due to the controversial use of ceftriaxone for treating infections caused by *Enterobacter* or *Citrobacter* species, the second sensitivity analysis retained patients with cultures positive for these species and excluded all other patients with a potentially ceftriaxone-intermediate or ceftriaxone-resistant infection.

Baseline characteristics collected included age, race/ethnicity, gender, height, weight, serum creatinine, source of infection, positive cultures and organisms, positive *Streptococcus pneumoniae* urinary antigen, SOFA score, and the presence of sepsis or septic shock. Body mass index (BMI) was calculated from each patient’s height and weight, and BMIs were then categorized using the Centers for Disease Control and Prevention’s definitions of underweight, normal weight, overweight, and obese [26]. The SOFA score assigns 0 to 4 points to six different organ systems based on the degree of dysfunction, with the total score ranging from 0 (no organ dysfunction) to 24 (maximal oral failure) [27]. For the purpose of calculating the SOFA score, normal values were assumed when the partial pressure of oxygen in arterial blood (PaO_2_) and the fraction of inspired oxygen (FiO_2_) were not available in the electronic health record.

Regarding ceftriaxone administration, the route of administration (IVP or IVPB), average daily dose, the duration of therapy, adverse drug reactions, escalation/change in therapy, change in ceftriaxone tolerability, and the total duration of all antibiotic therapy were collected. Adverse drug reactions and changes in tolerability were determined from provider notes in the electronic health record (EHR). Escalation or change in therapy was defined as a broadening of antibiotic therapy after ceftriaxone therapy had begun. De-escalation from ceftriaxone to an oral agent, such as cefdinir, was not considered to be an escalation of therapy. Additional data points included ICU LOS, total hospital LOS, all-cause ICU mortality, all-cause hospital mortality, and transition to comfort care or hospice.

The primary outcome of this study was treatment failure, defined as a composite of inpatient mortality and/or escalation of antibiotic therapy due to worsening clinical status as documented in the EHR [22]. If a patient had either outcome, they were considered to have a treatment failure. Secondary outcomes included escalation of therapy, ICU LOS, hospital LOS, ICU mortality, and hospital mortality.

All statistical analyses were conducted using IBM SPSS Statistics Version 29 (Armonk, NY, USA). Categorical and continuous outcomes were evaluated with the chi-squared and independent-sample *t*-test, respectively. Factors associated with treatment failure were evaluated using multivariate binary logistic regression. Variables considered in the model were determined *a priori* by consensus of the investigators and included IVP administration, the duration of therapy, SOFA score, and source of infection. The source of infection was categorized into respiratory, intra-abdominal including spontaneous bacterial peritonitis, urinary tract infection, severe infection (meningitis, endocarditis, pericarditis, or osteomyelitis), and other or unknown sources. All statistical tests were repeated for the two sensitivity analyses including the more limited patient groups. Missing data were excluded from all analyses, and *p*-values less than 0.05 were considered to be statistically significant.

## 5. Conclusions

In this retrospective, pre-/post-protocol change study, IVP administration of ceftriaxone compared to IVPB was associated with increased rates of treatment failure in critically ill patients. Future research should compare IVP and IVPB ceftriaxone for specific disease states, in different populations, and through a prospective, randomized study design. Until then, caution should be given to widespread IVP use of beta-lactam antimicrobials in critically ill patients until equivalent outcomes data are demonstrated compared to IVPB administration. When implementing novel antibiotic administration strategies in practice based primarily on convenience, preemptive consideration should be given to the impact of pharmacokinetics/pharmacodynamics, safety, and efficacy.

## Figures and Tables

**Table 1:** Baseline characteristics and interventions.

	IVP (*n* = 201)	IVPB (*n* = 200)	*p*-Value
Total Patients			
Age, years	61.7 ± 13.8	60.5 ± 16.3	0.458
Female gender	85 (42.3)	92 (46)	0.454
Body mass index, kg/m^2^	31.6 ± 9.4	30.6 ± 10.4	0.311
SCr, mg/dL at initiation	1.7 ± 1.7	1.8 ± 1.7	0.951
CrCl, mL/min at initiation	94.3 ± 75.3	86.7 ± 70.7	0.302
Race			
African American	55 (27.4)	43 (21.5)	0.437
Asian	0	1 (0.5)	
Caucasian	141 (70.1)	148 (74)	
Hispanic	4 (2)	3 (1.5)	
Organ Dysfunction			
SOFA score	6.4 ± 3.5	5.4 ± 2.9	0.002
Sepsis	113 (56.2)	61 (30.5)	<0.001
Septic shock	59 (29.4)	21 (10.5)	<0.001
Source of Infection			
Respiratory	107 (53.2)	80 (40.0)	0.023
Intra-abdominal/SBP prophylaxis	21 (10.4)	16 (8.0)	
Urinary tract infection	27 (13.4)	47 (23.5)	
Severe infection ^†^	22 (10.9)	25 (12.5)	
Other/unknown	24 (11.9)	32 (16.0)	
Interventions			
Duration of ceftriaxone, days	5.8 ± 2.4	5.9 ± 3.5	0.726
Average daily dose, g	1.33 ± 0.49	1.40 ± 0.91	0.335
Duration of antibiotic(s), days	10.3 ± 6.2	9.9 ± 5.9	0.538

All values are presented as numbers (percentages) or mean ± standard deviation. ^†^ Severe infection = meningitis, endocarditis, pericarditis, or osteomyelitis. IVPB = intravenous piggyback; IVP = intravenous push; SOFA = Sequential Organ Failure Assessment; SCr = serum creatinine; CrCl = creatinine clearance; SBP = spontaneous bacterial peritonitis.

**Table 2:** Primary and secondary outcomes.

Variable	IVP (*n* = 201)	IVPB (*n* = 200)	*p*-Value
Treatment failure	76 (37.8)	39 (19.5)	<0.001
Escalation of therapy	51 (25.4)	23 (11.5)	<0.001
All-cause hospital mortality	43 (21.4)	19 (9.5)	<0.001
All-cause ICU mortality	35 (17.4)	18 (9)	0.013
ICU length of stay, days	10.3 ± 9.2	9 ± 8	0.143
Hospital length of stay, days	18.6 ± 16.8	14.9 ± 10.2	0.009

All values are presented as numbers (percentages) or mean ± standard deviation. IVP = intravenous push; IVPB = intravenous piggyback; ICU = intensive care unit.

**Table 3:** Factors associated with treatment failure.

Variable	Odds Ratio	95% CI	*p*-Value
IV push administration	2.121	1.304–3.448	0.002
Duration of CTX therapy	0.875	0.792–0.968	0.009
SOFA score	1.191	1.106–1.283	<0.001
Source of infection			
Respiratory	Reference		
Intra-abdominal	0.817	0.341–1.955	0.649
Urinary tract infection	0.649	0.322–1.308	0.226
Severe infection	1.436	0.692–2.979	0.331
Other/unknown	0.756	0.355–1.613	0.470

All values are presented as numbers (percentages) or mean ± standard deviation. IV = intravenous; CTX = ceftriaxone; SOFA = Sequential Organ Failure Assessment.

**Table 4:** Baseline characteristics and interventions in sensitivity analyses.

Total Patients	Sensitivity Analysis #1: All Potentially Non-Susceptible Organisms Excluded			Sensitivity Analysis #2: *Enterobacter* and *Citrobacter* Species Retained		
	IVP (*n* = 176)	IVPB (*n* = 178)	*p*-Value	IVP (*n* = 184)	IVPB (*n* = 183)	*p*-Value
Age, years	61.6 ± 13.6	61.2 ± 16.0	0.797	62.0 ± 13.5	60.8 ± 16.3	0.432
Female gender	80 (45.5)	84 (47.2)	0.743	80 (43.5)	88 (48.1)	0.460
Body mass index, kg/m^2^	31.4 ± 9.3	30.7 ± 10.6	0.524	31.5 ± 9.5	30.5 ± 10.4	0.321
SCr, mg/dL at initiation	1.8 ± 1.7	1.8 ± 1.8	0.834	1.7 ± 1.7	1.8 ± 1.8	0.895
CrCl, mL/min at initiation	91.5 ± 73.3	83.5 ± 68.3	0.287	91.9 ± 72.7	85.4 ± 69.6	0.378
Race						
African American	45 (25.6)	40 (22.5)	0.670	49 (26.6)	41 (22.4)	0.606
Asian	0	1 (0.6)		0	1 (0.5)	
Caucasian	127 (72.2)	131 (73.6)		132 (71.7)	138 (75.4)	
Hispanic	3 (1.7)	2 (1.1)		3 (1.6)	3 (1.6)	
Organ Dysfunction						
SOFA score	6.4 ± 3.5	5.3 ± 2.8	<0.001	6.4 ± 3.5	5.3 ± 2.8	<0.001
Sepsis	99 (56.2)	51 (28.7)	<0.001	103 (56)	55 (30.1)	<0.001
Septic shock	52 (29.5)	17 (9.6)	<0.001	55 (29.9)	19 (10.4)	<0.001
Source of Infection						
Respiratory	93 (52.8)	68 (38.2)	0.029	97 (52.7)	71 (38.8)	0.021
Intra-abdominal/SBP prophylaxis	19 (10.8)	16 (9)		19 (10.3)	16 (8.7)	
Urinary tract infection	24 (13.6)	41 (23)		25 (13.6)	45 (24.6)	
Severe infection ^†^	20 (11.4)	23 (12.9)		21 (11.4)	23 (12.6)	
Other/unknown	20 (11.4)	30 (16.9)		23 (12.5)	32 (17.5)	
Interventions						
Duration of ceftriaxone, days	5.9 ± 2.3	5.9 ± 3.5	0.933	5.8 ± 2.4	5.8 ± 3.4	0.976
Average daily dose, g	1.3 ± 0.5	1.4 ± 0.9	0.427	1.3 ± 0.5	1.4 ± 0.9	0.474
Duration of antibiotic(s), days	10.2 ± 6.3	9.4 ± 5.6	0.239	10.3 ± 6.3	9.5 ± 5.5	0.174

All values are presented as numbers (percentages) or mean ± standard deviation. ^†^ Severe infection = meningitis, endocarditis, pericarditis, or osteomyelitis. IVPB = intravenous piggyback; IVP = intravenous push; SOFA = Sequential Organ Failure Assessment; SCr = serum creatinine; CrCl = creatinine clearance; SBP = spontaneous bacterial peritonitis.

**Table 5:** Primary and secondary outcomes in sensitivity analyses.

**Sensitivity Analysis #1: All Potentially Non-Susceptible Organisms Excluded**			
Variable	IVP (*n* = 176)	IVPB (*n* = 178)	*p*-Value
Treatment failure	60 (34.1)	33 (18.5)	<0.001
Escalation of therapy	42 (23.9)	18 (10.1)	<0.001
All-cause hospital mortality	35 (19.9)	17 (9.6)	0.006
All-cause ICU mortality	27 (15.3)	16 (9)	0.067
ICU length of stay, days	10.0 ± 9.0	8.2 ± 6.5	0.040
Hospital length of stay, days	18.5 ± 17.3	13.9 ± 9.2	0.002
**Sensitivity Analysis #2: *Enterobacter* and *Citrobacter* Species Retained**			
Variable	IVP (*n* = 184)	IVPB (*n* = 183)	*p*-Value
Treatment failure	66 (35.9)	34 (18.6)	<0.001
Escalation of therapy	45 (24.5)	19 (10.4)	<0.001
All-cause hospital mortality	39 (21.2)	17 (9.3)	0.001
All-cause ICU mortality	31 (16.8)	16 (8.7)	0.017
ICU length of stay, days	10.0 ± 9.0	8.4 ± 6.6	0.052
Hospital length of stay, days	18.5 ± 17.0	14.2 ± 9.3	0.003

All values are presented as numbers (percentages) or mean ± standard deviation. IVP = intravenous push; IVPB = intravenous piggyback; ICU = intensive care unit.

**Table 6:** Factors associated with treatment failure in sensitivity analyses.

**Sensitivity Analysis #1: All Potentially Non-Susceptible Organisms Excluded**			
Variable	Odds Ratio	95% CI	*p*-Value
IV push administration	1.855	1.087–3.164	0.023
Duration of CTX therapy	0.883	0.790–0.989	0.027
SOFA score	1.217	1.121–1.321	<0.001
Source of infection			
Respiratory	Reference		
Intra-abdominal	0.930	0.380–2.277	0.874
Urinary tract infection	0.883	0.424–1.836	0.738
Severe infection	1.714	0.787–3.734	0.175
Other/unknown	0.761	0.325–1.783	0.529
**Sensitivity Analysis #2: *Enterobacter* and *Citrobacter* Species Retained**			
IV push administration	2.036	1.209–3.430	0.008
Duration of CTX therapy	0.869	0.778–0.970	0.013
SOFA score	1.219	1.125–1.322	<0.001
Source of infection			
Respiratory	Reference		
Intra-abdominal	0.873	0.357–2.138	0.767
Urinary tract infection	0.777	0.377–1.601	0.495
Severe infection	1.715	0.797–3.687	0.168
Other/unknown	0.820	0.371–1.814	0.624

All values are presented as numbers (percentages) or mean ± standard deviation. IV = intravenous; CTX = ceftriaxone; SOFA = Sequential Organ Failure Assessment.

## Data Availability

Data are available upon reasonable request to the corresponding author.
